# Health professionals’ willingness to pay and associated factors for cervical cancer screening program at College of Medicine and Health Sciences, University of Gondar, Northwest Ethiopia

**DOI:** 10.1371/journal.pone.0215904

**Published:** 2019-04-30

**Authors:** Abebe Ayinalem Tarekegn, Mezgebu Yitayal Mengistu, Tsega Hagos Mirach

**Affiliations:** 1 Department of Human Anatomy, School of Medicine, College of Medicine and Health Sciences, University of Gondar, Gondar, Ethiopia; 2 Department of Health Service Management and Health Economics, Institute of Public Health, College of Medicine and Health Sciences, University of Gondar, Gondar, Ethiopia; Greenebaum Cancer Center, Institute of Human Virology, University of Maryland School of Medicine, UNITED STATES

## Abstract

**Introduction:**

Cervical cancer is a major public health problem in developing countries like Ethiopia. Cervical cancer screening service has been offered to high-risk groups in Ethiopia since 2013. However, there is no evidence on the willingness to pay for the screening. Therefore, we conducted this study to assess the female health professionals’ willingness to pay for cervical cancer screening at the College of Medicine and Health Sciences, University of Gondar, Northwest Ethiopia.

**Methods:**

Institutional based cross-sectional study design was used to assess the health professionals’ willingness to pay for the cervical screening from March to April, 2018. Simple random sampling technique was used to select study participants from a list of female health professionals who has been working for the College of Medicine and Health Sciences, University of Gondar. The data were entered into EpiData version 3.1 and exported to STATA version 14 for analysis. Tobit models were used to identify factors which had statistical significant association with willingness to pay for cervical cancer screening service.

**Results:**

A total of 392 respondents participated in the study with a response rate of 92.7%. The majority (83.4%) of participants were willing to pay for cervical cancer screening. The average amount of money they were willing to pay was ETB 194.7 (US$7.16) per service. Age ≥ 30 years, educational status, perceived seriousness of cervical cancer, perceived quality of cervical cancer screening service and monthly income were significantly associated with willingness to pay for cervical cancer screening.

**Conclusion:**

High proportion of study participants were willing to pay for cervical cancer screening. Therefore, the policy makers can scale-up cervical cancer screening by setting appropriate fee for service charge. They can also raise awareness of cervical cancer and offer quality service in order to increase the benefits of the program.

## Introduction

Cervical cancer is a chronic disease that arises due to the abnormal growth of cells from the lower part of the uterus which projects to the vagina [1]. Human Papilloma Virus (HPV) accounts for more than 99% of all cervical cancer cases [2]. Persistence of HPV infection of the cervix is a necessary cause of cervical cancer. Immune suppression, multiple sexual partners, co-infection with other sexually transmitted agents, Human Immunodeficiency Virus (HIV), multiparity, family history of cervical cancer and tobacco smoking are risk factors for persistence of HPV infection of cervix [3–5].

Global incidence rates of cervical cancer is about 500,000 cases each year [6] and Sub-Saharan African countries account for 22% of all cases in the world [7]. Cervical cancer causes 266,000 women to die each year in the world [3]. In Eastern Africa, the incidence and mortality rate of cervical cancer per 100,000 women were estimated to be 42.7 cases and 35 deaths, respectively [3, 4, 8, 9] while, in Ethiopia, the incidence and mortality rate of cervical cancer were estimated to be 26.4 and 18.4 per 100,000 women, respectively [10].

Deaths (lost years of life) and disability (lost productivity) associated with all types of cancer cause loss of 1.5% global gross domestic product (GDP) without including direct medical cost of the treatment. In High- and Middle-income countries, most of this loss was due to lung, colon/rectal, and breast cancers. However, in 2008 Low-income countries economic loss to cancer was due to mouth and throat, cervix, and breast cancers which incurred 1.3US$ billion, 1.3US$ billion and 1.1US$ billion respectively [11].

Cervical cancer is preventable by reducing risk factors and by screening vulnerable groups. Many countries in the world implement cervical cancer screening program as their first strategy to reduce cervical cancer disability and mortality [3]. Cervical cancer screening was introduced to developing countries, including Ethiopia, recently.

Cervical cancer screening is a process of detecting precancerous cells before a woman develops any symptoms of cervical cancer [1]. Cervical cancer screening by itself does not prevent cervical cancer but screening is a gateway to link those who are positive to precancerous cells to treatment [3, 5].

Cervical cancer screening program was started in different sites in Ethiopia with support from donors in 2013.

The rate of cervical cancer screening utilization is among female nurse professionals in public health institutes in Mekelle town in Ethiopian [12] which was lower than women utilization rate in Mekelle Zone which was 19.8% [13]. There has been no previous study of female health professionals willingness to pay (WTP) for cervical cancer screening, hence this study.

The result of this study may help policy makers devise strategies to scale up cervical cancer screening program based on the female health professionals’ willingness to pay for the program. In addition, the study may also help serve to estimate the fees, in a fee-for-service model.

## Methods

### Study design and settings

Institutional based cross-sectional study was conducted to assess the female health professionals’ willingness to pay for cervical cancer screening at the College of Medicine and Health Sciences, University of Gondar. The University of Gondar is a higher institution in Ethiopia located in Northwest part of the country at historical Gondar town which is 750 km away from Addis Ababa. The College of Medicine and Health Sciences (CMHS), is one of the seven campuses in the University of Gondar. CMHS campus has a hospital, known as University of Gondar Specialized Referral Hospital (UoGSRH), which serves as teaching hospital and also provides health care services. The College had 852 female hospital staff and 166 female academic staff during the data collection period. The study was conducted from March 1^st^—April 30^th^, 2018.

### Study population and sampling

All female health professionals who were working at CMHS were the study population. Those health professionals who had sick leave and pregnancy leave were excluded from this study during data collection.

The sample size of the study was calculated by using single population proportion formula for willingness to pay using the Epi Info^™^ Stat Calc program with the assumption of 50% willingness to pay (p), 95% confidence level, 5% margin of error, and 10% response rate.

$$n=\frac{\left(Za/2)2*p*(1-P\right)}{W2}$$

where p = 50%, margin of error (*w)* = 0.05.

Therefore, the final sample size of the study was 423 female health professionals.

The study participants were selected randomly by lottery method from the list of study population which was accessed from the UoGSRH Human Resource Management Department and CMHS Human Resource Management Department.

### Data collection procedures

An interviewer administered questionnaire was used to collect the data. The questionnaire consists of questions on sociodemographic characteristics, knowledge related characteristics, health and service related factors, economic characteristics of the study participants, and hypothetical scenario about cervical cancer screening service for willingness to pay. Double-Bounded Dichotomous Choice Variant of the Contingent valuation (CV) method was used to estimate the benefit of the program by eliciting the WTP of female health professionals. This method was used to estimate the benefit of the program in terms of monetary unit. The study participants were asked the maximum amount they were willing to pay for the service they got per visit. The questionnaire was pre-tested on 32 female health professionals who were working at Filege Hiwot Referral Hospital which is located in the same region, Amhara National Regional State, Ethiopia.

### Operational definitions

#### Willingness to pay

This is the maximum price the study participant will definitely be willing to pay per unit of a service. The mean amount money the study participants were willing to pay was calculated as follows:

$$AMWTP=\sum_{n=1}^{392}(MWTP1+MWTP2+\cdots \ldots \ldots ..+MWTP545)÷392$$

#### Contingent value method

This is an approach where the study participant was first asked whether they would be willing to pay a specific amount per service and then the question was repeated using a higher or lower bid value depending on the response to the first question until reaching to the final maximum amount the study participant is willing to pay. The initial price set to elicit the preference of the study participants was 200 ETB. The exchange rate during data collection was 1 USD (United States Dollar) = 27.2 ETB (Ethiopian Birr).

#### Knowledge about cervical cancer

This is any information about signs, symptoms and risk factors for cervical cancer and it was measured by 12 knowledge items with responses of “Yes which was scored as 1”, “No and I do not know” which was scored as 0”. The total knowledge scores was interpreted based on an interquartile, whereby the first quartile (25%) who scored ≤ 3 was interpreted as “Poor knowledge”, the second and third quartiles (>25% and <75%) who scored >3 and < 9 was interpreted as “Moderate Knowledge” and the last quartile (75%) who scored > or equal to 9 was interpreted as “Excellent Knowledge” about cervical cancer.

#### Perceived quality of cervical cancer screening service

This is the extent of female health professionals perception of the quality of cervical cancer screening service delivery and it was measured by one item 5-points Likert scale questions.

#### Perceived seriousness of cervical cancer

This is the extent of female health professional’s perception of the seriousness of cervical cancer and it was measured by one item 3-points Likert scale questions.

#### Health status

This is the extent of female health professionals feeling on their own health and evaluated as good, medium or poor.

### Data processing and analysis

The data was entered into EpiData version 3.1 and exported to STATA version 14 for analysis. The data was described using frequencies and means to show distribution of the outcome variable and associated factors. Tobit model was used to analyse the determinants of willingness to pay and the maximum amount of money that individuals were willing to pay for cervical cancer screening. This model has an advantage over other discrete choice logistic models in that, it reveals the maximum amount of money the respondents were willing to pay.

$$Y=1\;if\;MWTP=\beta o+\beta^{\prime }Xi+\varepsilon >0\;and\;Y=0\;if\;MWTP\leq 0$$

Where

Y = Outcome variable; MWTP = Maximum WTP; Xi = Explanatory variables; βo = Slope; β’ = Coefficient; ε = error term; 1 = Success/Yes; 0 = Failure/No

The model estimates marginal effect of an explanatory variable on the expected value of the outcome variable. Normality and equal variance assumption of Tobit model were checked by Kdensity and Pnorm tests. A p-value = 0.05 was used to determine statistical significance.

### Ethical considerations

Ethical clearance (Ref No/IPH/271/2017) was obtained from the Ethical Review Committee of the Institute of Public Health, College of Medicine and Health Sciences, University of Gondar. To obtain participants consent; the purpose and importance of the study was explained in the consent form, including the right to withdraw from the study if they face any inconvenience. Name of respondent was not included in the questionnaire and the confidentiality of the data was protected at all levels.

## Results

### Socio-demographic and economic characteristics of the respondents

A total of 392 study participants, with a response rate of 92.7%, were included in the study. The mean age of the respondents was 28 years. The majority of respondents were Orthodox Christians (318(81.3%)) and Amhara (347(88.5%)), Unmarried (242(71.4%)), first degree holders (277(71.2%)) and nurses (187(47.2%)) (Table 1). On the average, monthly income of participants was 6177.47ETB.

**Table 1:** Demographic and socioeconomic characteristics of study participants, CMHS, UoG, Ethiopia, 2018 (N = 392).

Variable	Description	Frequency (%)
Religion of the respondent	Orthodox Christians	318(81.3%)
	Muslim	53(13.6%)
	Protestant	17(4.35%)
	Others	3(0.77%)
Ethnicity of the respondent	Amhara	347(88.5%)
	Tigre	24(6.12%)
	Oromo	10(2.55%)
	Others	11(2.81%)
Marital status of the respondent	Unmarried	242(61.7%)
	Married	136(34.7%)
	Divorced	11(2.81%)
	Widowed	3(0.77%)
Background professional specialities of the respondent	Nurse	185(47.2%)
	Midwifery	79(20.2%)
	Public Health officer	45(11.5%)
	Pharmacy	44(11.2%)
	Medical laboratory	23(5.87%)
	Others	16(4.08%)
Educational status of the respondent	Diploma	73(18.6%)
	First Degree	277(70.7%)
	Second Degree	42(10.7%)

### Health status and services related characteristics of study participants

Around three-fifth (225(57.4%)) of the respondents had the high perceived quality of the cervical screening service and 222(56.6%) of respondents said they had a good health status (Table 2).

**Table 2:** Health status and services related characteristics of the study participants, CMHS, UoG, Ethiopia, 2018.

Variable	Description	Frequency (%)
Health status of participants(n = 392)	Poor	34(8.7%)
	Medium	136(34.7%)
	Good	222(56.6%)
Perceived quality of the service(n = 392)	Very Low	12(3.06%)
	Low	60(15.3%)
	Medium	42(10.7%)
	High	225(57.4%)
	Very High	53(13.5%)

### Knowledge of study participants on cervical cancer and its screening program

In this study, 384(98%) of respondents had heard about cervical cancer screening. Among sources of information, schools were the main source for 233(60.7%) respondents while 269(68.6%) of the respondents had excellent knowledge about cervical cancer symptoms and its risk factors (Table 3).

**Table 3:** Health professionals’ knowledge for cervical cancer and its screening at CMHS, UoG, Ethiopia, 2018.

Variable	Description	Frequency %
Have you heard about cervical cancer screening? (n = 392)	Yes	384(98%)
	No	8(2.04%)
What is your source of more information(n = 384)	School	233 (60.7%)
	TV/Radio	101 (26.3%)
	Training	33 (8.59%)
	Internet	14 (3.65%)
	Other	3 (0.78%)
Perceived seriousness of cervical cancer(392)	Less serious	52 (13.3%)
	Serious	227 (57.9%)
	Highly serious	113 (28.8%)
Knowledge of respondents (n = 392)	Poor Knowledge	27 (6.89%)
	Moderate Knowledge	96 (24.5%)
	Excellent Knowledge	269 (68.6%)

### WTP for cervical cancer screening service

Among participants in this study, 327(83.4%) were willing to pay for cervical screening service. The mean amount of money female health professionals were willing to pay was 194.7 ±132 ETB, and 113(34.6%) of study participants were willing to pay more than 300 ETB (Table 4).

**Table 4:** Amount of money female health professionals were willingness to pay for cervical cancer screening service, CMHS, UoG, Ethiopia, 2018.

Variable	Description	Frequency %
Health professional willingness to pay for cervical cancer (n = 327)	10–100 ETB	45(13.8%)
	101–150 ETB	39(11.9%)
	151–200 ETB	54(16.5%)
	201–250 ETB	46(14.1%)
	251–300 ETB	30(9.2%)
	>300 ETB	113(34.6%)

### Factors associated with willingness to pay for cervical cancer screening service

The study showed that age ≥ 30years, perceived seriousness of cervical cancer, perceived quality of cervical screening service, educational status and monthly income were factors significantly associated with willingness to pay for cervical cancer screening (Table 5).

**Table 5:** Factors associated with WTP for cervical cancer screening service, CMHS, UoG, Ethiopia, 2018.

Parameter for MWTP	Category	Coefficient	Standard error	t-value	P-value	95% Confidence Interval	Marginal effect = Probability (dy/dx)
Age(ref. age<30 years)	D						
Age≥30years		-25	10.6	-2.24	0.019 *	-45.9, -4.2	-.025
Marital status(ref. married)	S						
Single		-10.7	10.9	-0.98	0.325	-32.1, 10.7	
Divorced		2.4	32	0.07	0.940	-60.5, 65.3	
Widowed		16	50.8	0.31	0.753	-84, 116	
Religion (ref. Orthodox)	S						
Muslim		0.12	15.2	0.01	0.994	-29.8, 30.1	
Protestant		-16.9	25.4	-0.66	0.507	-66.8, 33.1	
Others		-42.4	60.9	-0.70	0.487	-162.3, 77.5	
Ethnicity	S						
Tigre		7.7	18.5	0.42	0.678	-28.7, 44.1	
Oromo		2	33.5	0.06	0.954	-64, 67.8	
Others		-8	27.5	-0.29	0.770	-62.1, 46.1	
Background profession (ref. Public Health Officer)	S						
Pharmacy		9.9	19.7	0.50	0.618	-28.9, 48.6	
Nurse		-0.9	15.4	-0.06	0.953	-31.2, 29.4	
Midwifery		11.1	17.5	0.63	0.527	-23.3, 45.5	
Medical Laboratory		3.1	27.2	0.11	0.909	-50.4, 56.5	
Others		-2.5	25.8	-0.10	0.922	-53.2, 48.2	
Educational status(ref. Diploma)	S						
First Degree		33	17.9	1.85	0.066	-2.2, 68.3	
Second Degree		48.6	24.5	1.99	0.048*	0.45, 96.8	.01
Knowledge (ref. poor Knowledge)	S						
Moderate Knowledge		19.2	23.8	0.81	0.421	-27.6, 66	
Excellent Knowledge		19.2	22.1	0.87	0.386	-24.3, 62.6	
Health Status(ref. Poor	S						
Medium		-20	20.4	-0.98	0.327	-60.1, 20.1	
Good		-24.3	19.2	-1.26	0.207	-62.1, 13.6	
Perceived seriousness cervical cancer (ref. less serious)	S						
Serious		121.2	17.5	6.94	0.000 ***	86.9, 155.6	.056
Highly serious		139.2	18.7	7.44	0.000 ***	102.4, 176	
Perception of service quality (ref. Very Low)	S						
Low		115	52.4	2.19	0.029*	11.8, 218.3	
Medium		179.3	52	3.44	0.001***	76.8, 281.7	.059
High		198	50.4	3.93	0.000***	98.8, 297	
Very High		228	51.8	4.40	0.000***	126, 330	
Source of more Information (ref. Internet)	S						
Mass media		-10.4	26	-0.40	0.690	-61.6, 40.8	
School		-5.4	25.3	-0.21	0.832	-55.1, 44.4	
Training		6.6	30.9	0.22	0.830	-54.1, 67.4	
Others		37.3	54.5	0.68	0.495	-70, 144.6	
Monthly Income(Birr)	N						
		**0.04**	**0.004**	**11.3**	0.000***	**0.03, 0.05**	**.00005**

**Note**:

***significant with p-value ≤ 0.001;

*significant with p-value ≤ 0.05; S = String variable; N = Numeric value and D = Dummy Variable

Respondents whose ages were ≥30 years were willing to pay 25 ETB (2.5%) less compared to respondents whose ages were < 30 years. (P = 0.019, average marginal effect = -0.025).

Second degrees were willing to pay 48.60 ETB (1%) more compared to those who held diploma holders (P = 0.048, average marginal effect = 0.01).

The study indicated that health professionals who had low, medium, high and very high perceived quality of cervical cancer screening were willing to pay 115.00 ETB, 179.30 ETB, 198.00 ETB, and 228.00 ETB more compared to health professionals who had very low perceived quality of screening service. Participants who had medium and above medium perceived quality service were willing to pay 5.9% more than those respondents who had low perceived quality service (P = 0.000, average marginal effect = 0.059).

The participants who perceived cervical cancer as a serious and highly serious disease were willing to pay 121.20 ETB and 139.20 ETB more compared to participants who perceived it as a less serious disease. Participants who perceived cervical cancer as a serious disease were willing to pay 5.6 more compared to those who perceived cervical cancer as less serious disease (P = 0.000, average marginal effect = 0.056).

As monthly income increased by one Birr, the willingness of the respondents to pay increased by 0.04 ETB. A monthly income of the participants increased by 1000.00 ETB, 5% of the respondents became more willing to pay for cervical cancer screening service (P = 0.000, average marginal effect = 0.00005) (Table 5).

## Discussion

The study showed that 83.4% of the respondents were willing to pay for cervical cancer screening service. We also found that age ≥30 years, possession of a degree, perception of the quality of cervical cancer screening service is high, that cervical cancer is a serious illness and increasing income were associated with the female health professionals willingness to pay for cervical cancer screening service.

The proportion of female health professionals WTP for cervical cancer screening was somewhat higher than those willing to pay for injectable contraceptive and foot wear for Podoconiasis which were (68%) and (72.8%) respectively in studies done in Tigray and Northern Ethiopia [14, 15]. This might be due to study population differences in information about health. However, the result of the study was lower than WTP of respondents for health service in Iran which was 88.8% [16]. The possible reason might be difference in study areas, cultural, demographic and study population difference.

The average amount of money that respondents were willing to pay for cervical cancer screening service was 194.70 ETB (US$7.16) which was 5.00 ETB less than the average price set to elicit preference. The average amount of money the respondents were willing to pay for cervical cancer screening was much higher than what respondents were willing to pay for injectable contraceptive and foot wear for Podoconiasis per year which were 11.00 ETB (US$0.65) and 64.00 ETB (US$ 3.28) [14, 15]. This may be due to population difference, the currency inflation, differences in preventive health services studied and the target population appreciation of the usefulness or importance of different preventive health services. However, the result of this study was lower than a study done on WTP for HPV vaccine which was US$ 11.68 in Nigeria [17] and US$ 50.26 in Malaysia [18], and WTP for the clinical preventive services which was US$ 93.72 in South Nigeria [19]. This may be due to differences in socio-status of study participants and program preference.

Respondents with age ≥ 30years were less willing to pay for cervical cancer screening services. This may be because respondents’ age ≥ 30 years have lower family income than younger age groups. Other studies done on WTP for Pap test in Nigeria [20], for health care services in Malaysia [21] and Iran [22] had a positive association. This might be because population in Nigeria, Malaysia and Iran had good health seeking behavior. The result also contradicted with studies done on WTP for mortality risk reduction from the environment in USA [23]. One possible explanation for this difference may be that all age groups perceived themselves to be equally at risk from environmental factors.

This study also showed that educational status was significant for WTP for cervical cancer screening service. This may be because as educational status increases, individuals have more information and are more aware about their health issues. This study is in line with studies done in different parts of the world such as Addis Ababa [24], Malaysia [21], Iran [22].

The knowledge of health professionals’ about cervical cancer and its risk factors did not have statistical association with willingness to pay. This finding is different from other WTP studies conducted Ethiopia [25], USA [26]. The possible explanation may be all participants were health professionals and selected from the same study area.

The multivariable analysis revealed that perceived seriousness of cervical cancer had a positive association with willingness to pay for cervical cancer screening service. The reason for this might be peoples want to prevent themselves from high-burden diseases. This is supported by studies done on willingness to pay for medical care among government school teachers in Addis Ababa [24], willingness to pay for depression treatment USA [27].

The respondents who had low, medium, high and very high perceived quality of cervical screening service were willing to pay more than respondents who had very low perceived quality. This may be because individuals are aware of quality service which requires more money. This result is consistent with studies done on WTP for Alzheimer’s disease prevention measures in USA [28] and willingness to pay for health care services in Malaysia [21].

The monthly income of the respondents’ had significant association with WTP for cervical screening service. This may be due to those having more income may had additional money to allocate for promotion of their health in addition to meeting their basic needs. Studies conducted in Addis Ababa [24], Malaysia [21] Iran [16], Korea [29] and USA [28] for different health care services support this conclusion.

### Limitation of the study

The method used in this study is partial economic evaluation approach which does not show net benefit of the cervical cancer screening service since the study did not account for other costs and benefits of cervical cancer prevention methods. The study only considered health professionals and is not generalizable to the population. The other limitation of the study might be response bias in which respondents intentionally prefer lower costs of cervical cancer screening service to ensure that the set price is low when currently free services end.

## Conclusion and recommendation

The study showed delivering quality service to customers, focusing on raising awareness will further strengthen the demand for cervical cancer screening service. Qualitative study should be done on the benefit of cervical cancer screening program since there is a gap between proportion of cervical cancer screening service utilization and willingness to pay.

## Supporting information

S1 File (DOCX)

S1 Data (DTA)
