# Supplementary material for: Health professionals’ willingness to pay and associated factors for cervical cancer screening program at College of Medicine and Health Sciences, University of Gondar, Northwest Ethiopia
Source: PLoS One. 2019 Apr 30;14(4):e0215904. doi: 10.1371/journal.pone.0215904 (PMC6490889; doi:10.1371/journal.pone.0215904)
Supplement: S1 File — (DOCX) [file pone.0215904.s001.docx]

## Questionnaire

Questionnaire No____________

| PART 1- SOCIO-DEMOGRAPHIC CHARACTERISTICS | | | | |
| --- | --- | --- | --- | --- |
| Code | QUESTION | RESPONSE CODE (CIRCLE THE APPROPRIATE OPTION OR ENTER NUMBERS) | | SKIP |
| 101 | Age of the respondent(years) | Age ----------------------- | |  |
| 102 | Marital Status of the respondent | Married----------1 Single-----------3  Widowed--------2 Divorced--------4  Others----------96 | |  |
| 103 | Religion of respondent | Orthodox-------1 Muslim------------------------3 Protestant-------2 Others(specify)-------------96 | |  |
| 104 | Ethnicity of respondent | Amhara--------------------1  Tigre------------------------2  Oromo----------------------3  Others(specify)------------96 | |  |
| 105 | Educational status of respondent | Diploma----------------------------------1  First Degree------------------------------2  Second Degree---------------------------3  Third Degree-----------------------------4 | |  |
| 106 | The respondent health profession background | Medical Doctor-----1 Midwifery---------5  Heath Officer-------2 Anaesthesia -------6  Pharmacy------------3 Laboratory---------7  Nurse-----------------4 Other(Specify)-----96 | |  |
| PART 2 –KNOWLEDGE RELATED QUESRIONS | | | | |
| No | Question | | Response |  |
| 201 | Have you heard about Cervical cancer screening? | | Yes-------1  No---------2 | If you say yes go to 202 |
| 202 | What is your main source of information about cervical cancer screening? | | Internet------------------1  Mass media (Television, Radio)------------------2  Schools-----------------3  Newspaper-------------4  Training-----------------5  Other source------------6 |  |
| 209 | Do you know about the ***dangers signs of Cervical Cancer?*** | | Yes-----------------------1  No------------------------2 | If you say yes, go to 210-213 |
| 210 | Is **Pain/discomfort during intercourse** a danger sign of Cervical cancer? | | Yes -----------------------1  No-------------------------2  You don’t know---------3 |  |
| 211 | Is ***vaginal bleeding*** a danger sign of Cervical cancer? | | Yes------------------------1  No-------------------------2  You don’t know---------3 |  |
| 212 | Is ***foul smelling vaginal discharge*** a danger sign of Cervical cancer? | | Yes------------------------1  No-------------------------2  You don’t know---------3 |  |
| 213 | Is ***anemia*** a danger sign of Cervical cancer? | | Yes------------------------1  No-------------------------2  You don’t know---------3 |  |
| 214 | Do you know the ***risk factors for cervical cancer?*** | | Yes------------------------1  No-------------------------2 | If you say yes, go to 215-222 |
| 215 | Is ***Human Papilloma Virus*** a risk factor for cervical cancer? | | Yes------------------------1  No-------------------------2  You don’t know---------3 |  |
| 216 | Is ***multiparty*** a risk factor for cervical cancer? | | Yes------------------------1  No-------------------------2  You don’t know---------3 |  |
| 217 | Is ***immunosuppressed*** a risk factor for cervical cancer? | | Yes------------------------1  No-------------------------2  You don’t know---------3 |  |
| 218 | Is ***early mirage < 18 years*** a risk factor for cervical cancer? | | Yes------------------------1  No-------------------------2  You don’t know---------3 |  |
| 219 | Is ***family history (Genetics)*** a risk factor for cervical cancer? | | Yes------------------------1  No-------------------------2  You don’t know---------3 |  |
| 220 | Is ***smoking*** a risk factor for cervical cancer? | | Yes------------------------1  No-------------------------2  You don’t know---------3 |  |
| 221 | Having ***multi sexual partner*** is a risk factor for cervical cancer? | | Yes------------------------1  No-------------------------2  You don’t know---------3 |  |
| 222 | Is cervical cancer preventable disease? | | Yes------------------------1  No-------------------------2  You don’t know---------3 |  |
| 223 | What other risk factors do you know for cervical cancer? ----------------------- | |  |  |
| 224 | How serious do you think cervical cancer is? | | Less serious--------------1  Serious-------------------2  Highly serious-----------3 |  |

| PART 3- Health and health related questions | | | |
| --- | --- | --- | --- |
| Q. | QUESTION | RESPONSE CODE (CIRCLE THE APPROPRIATE OPTION OR ENTER NUMBERS) | SKIP |
| 300 | How you rate your health status? | Poor------------------------1  Medium--------------------2  Good-----------------------3 |  |
| 301 | Have you screened for cervical cancer? | Yes--------------------------1  No---------------------------2 |  |
| 302 | How do you rate your perception on the quality of cervical cancer screening service? | Very Low-------------------1  Low-------------------------2  Medium---------------------3  High-------------------------4  Very High-------------------5 |  |

**PART 4:- INCOME QUESTIONS**

| 401 | What is your salary? | ----------- Birr |
| --- | --- | --- |
| 402 | Do you have other source of income? | Yes---------1  No----------2 |
| 403 | If say yes question number 402, how much do get monthly your other source of income? | --------- Birr |
| 403 | How much is your family income? | -----------Birr |
| 404 | Do you have bank account? | Yes----------1  No----------2 |
| 404 | How much you have in your/your family account? | ------------Birr |

| PART 5:CASE SCENARIO ABOUT CERVICAL CANCER SCREENING  **Introduction:** Cervical cancer is one of the major existing global problems. Even if this problem affects global community, the significance is very high in developing countries. The burden of the problem is highly affects females. The problem in this segment of the population causes a devastating impact. Different evidences shows that the cause of this problem is mainly due to knowledge related problems related to cervical cancer prevention practice. To solve this problem different prevention are designed by different countries of the world. The Ethiopian government has been design cervical cancer screening strategy to prevent the incidence of cervical cancer. This program has been implemented by health professionals.  **Here is cervical cancer screening and its benefits:**  ***Cervical cancer screening program*** has different benefits for the community. Among the benefits counseling about prevention, risk factors and treatment of cervical cancer. This ***Cervical cancer screening service*** the most effective method among cervical cancer prevention methods after takes a sample from cervix. The project is given by the fund of government and other partners. based on this scenario if this service was given with fee … |
| --- |

1. Are you willing to pay for the above service? Yes......1

No.......2

1. If “yes” continue by below diagram:

Max=? ____

300ETB

YES

YES

NO

250 ETB

No

Max=? _____

YES

Max=? _______

Are you willing to pay ***200ETB*** for cervical screening service?


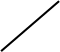


NO

150ETB

Max =? ______

YES

Max=? _____

NO

NO

100ETB

Max=? _______

YES

***NB***: ETB = Ethiopian Birr

**Thank you being a part of this study!!!!!!!**
